# PD-1 and PD-L1 expression in 132 recurrent nasopharyngeal carcinoma: the correlation with anemia and outcomes

**DOI:** 10.18632/oncotarget.17214

**Published:** 2017-04-19

**Authors:** Yajuan Zhou, Jingjing Miao, Haijun Wu, Hao Tang, Jing Kuang, Xiaoyi Zhou, Yi Peng, Desheng Hu, Dingbo Shi, Wuguo Deng, Xinyue Cao, Chong Zhao, Conghua Xie

**Affiliations:** ^1^ Hubei Key Laboratory of Tumor Biological Behaviors, Department of Radiation and Medical Oncology, Zhongnan Hospital of Wuhan University, Wuhan, China; ^2^ Department of Radiation Oncology, Hubei Cancer Hospital, Wuhan, China; ^3^ Department of Nasopharynx, Collaborative Innovation Center for Cancer Medical, State Key Laboratory of Oncology in South China, Sun Yat-Sen University Cancer Center, Guangzhou, China; ^4^ Department of Pathology, Hubei Cancer Hospital, Wuhan, China; ^5^ Collaborative Innovation Center for Cancer Medical, State Key Laboratory of Oncology in South China, Sun Yat-Sen University Cancer Center, Guangzhou, China; ^6^ Department of Biological Repositories, Zhongnan Hospital of Wuhan University, Wuhan, China

**Keywords:** PD-L1, PD-1, HIF-1α, nasopharyngeal carcinoma, recurrent

## Abstract

The expression of Programmed death-1 (PD-1) / programmed death-ligand 1 (PD-L1) has been reported to be reliable prognostic factors in various malignances including primary nasopharyngeal carcinoma (NPC). However, the exact role of PD-1/PD-L1 in recurrent NPC remains unclear. In this study, we aimed to investigate the relationship between the expression of PD-1 / PD-L1 and the clinical-pathology as well the outcomes of recurrent NPC patients (n = 132). The expression of PD-1 and PD-L1 was measured by immunohistochemistry staining. The relationship between PD-1 / PD-L1 and factors involved in clinic-pathology and outcomes of patients with NPC was assessed by correlation analysis. To further explore the association between PD-L1 and anemia, immunofluorescence analysi*s* was performed to investigate the correlation of PD-L1 with hypoxia inducible factor-1α (HIF-1α). We observed that advanced rT classification and anemia status before salvage treatment was associated with high level of PD-L1 in recurrent NPC patients, and PD-L1 and was co-located with HIF-1α in recurrent tumors by immunofluorescence analysis. Moreover, our result suggested that PD-L1 might be a negative indicator for recurrent NPC patients as well as age, rT classification, anemia and tumor necrosis at diagnose of recurrence. Taken together, our results revealed that PD-L1 might be a potential prognostic biomarker for recurrent NPC patients, and advanced re-stage, anemia might represent as candidate biomarkers for evaluating patients’ response to anti-PD-1 / PD-L1-treatment. However, further studies are needed to clarify the underlying mechanism of hypoxia in immunosuppression process induced by PD-1 / PD-L1 axis.

## INTRODUCTION

Nasopharyngeal carcinoma (NPC) is endemic in southern China, where annual incidence ranges from 20 to 50 cases per 100,000 [1]. The local control of NPC was significantly improved due to advances in radiotherapy and combined chemotherapy, however, approximately 10% patients with NPC develop local and regional recurrences [2, 3]. Recurrent NPC has a unique profile of pathological and clinical features [4, 5]. Traditional salvage treatments offer limited clinical benefits and often cause severe complications. Although several biomarkers were identified for evaluating the prognosis of recurrent NPC, the overall survival (OS) rate of patients is still not improved with 5-year OS rate being only 30% [6, 7], making the management of recurrent NPC being a big challenge in clinic [8, 9]. Therefore, seeking reliable prognostic markers as well as effective treatments are urgently required.

Programmed death-1 (PD-1 or CD279) is a co-receptor expressed predominantly by T cells [10]. Programmed death-ligand 1 (PD-L1 or CD274) engaged by PD-1 in cancer cells could inhibit the self-reactivation of T cells and induce tumor immune tolerance [11]. The predictive value of PD-1 / PD-L1 has been recognized in multiple tumors [12–15], including primary NPC. However, the prognostic role of PD-1 and PD-L1 in NPC were controversy. For instance, our previous study [16] suggested that PD-L1 was correlated with worse outcome of primary NPC patients. Zhang *et al* [17] reported the similar negative correlation between PD-L1 and disease-free survival of NPC. In contrast, Lee *et al* [18] delineated PD-L1 expression was positively correlated with longtime survival of NPC. Hsu *et al* [19] has shown that PD-1 on CD8 T cells predicted poor prognosis in 48 NPC patients. Interestingly, PD-1 expression might not be a prognostic factor in NPC patients according our former study [16] and Zhang *et al* [17]. In addition to the conflicting findings in primary NPC, the prognostic value of PD-1 / PD-L1 in recurrent NPC has been rarely reported.

Therapies targeting the PD-1 / PD-L1 pathway show promising results in the treatment of cancers through promoting antitumor T-cell activity [20, 21]. There are also several ongoing clinical trials evaluating PD-1 / PD-L1 checkpoint blockades in the treatment of NPC [22]. The overexpression of PD-1 / PD-L1 is presented as most effective predictive marker for evaluating patients’ response to PD-1 / PD-L1 antibodies treatment [23, 24]. However, tumor microenvironment such as tumor hypoxia [25] might also account for the heterogeneous responses during immunotherapies. A previous study [26] demonstrated that PD-L1 is a direct target of hypoxia-induced factor-1α (HIF-1α) and PD-L1 expression was up-regulated under hypoxia. In addition, blockage of PD-L1 enhanced myeloid-derived suppressor cells-mediated T cell activation, suggesting simultaneous blockage of PD-L1 and HIF-1α might represent a novel approach for cancer immunotherapy.

In this study, we aimed to investigate the prognostic role of PD-1 and PD-L1 in a cohort of recurrent NPC patients (n = 132). In addition, we also evaluated the correlation between PD-1 / PD-L1 expression and clinic-pathological variables as well as potential related peripheral blood biomarkers, such as hemoglobin (HB) level. Our study revealed that PD-L1 might be a potential prognostic biomarker for recurrent NPC patients, and advanced re-stage, anemia might represent as candidate biomarkers for evaluating patients’ response to anti PD-1 / PD-L1-treatment.

## RESULTS

### General information and survival outcomes

**Figure 1:**
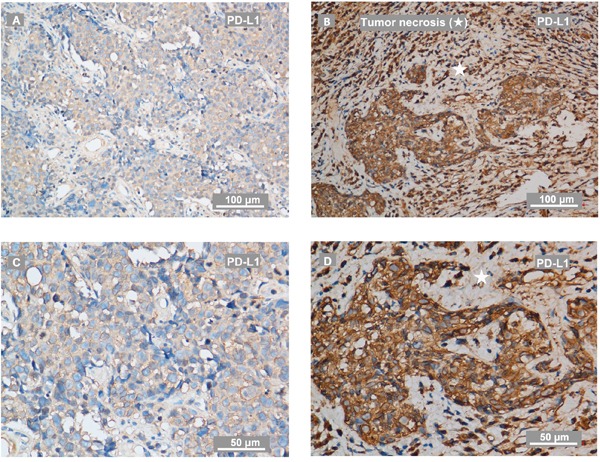
Representative IHC staining of PD-L1 and tumor necrosis on recurrent NPC-biopsies **(A, C)** PD-L1 staining on biopsy from a patient with recurrent NPC evaluated as low level of PD-L1. The IHC photos were taken by a phase-contrast microscopy and exhibited as low **(A)** and high magnification **(C)**. **(B, D)** PD-L1 staining on biopsy from a patient with recurrent NPC evaluated as high level of PD-L1. The IHC photos were exhibited as low **(B)** and high magnification **(D)**. PD-L1 expressed in tumor-infiltration lymphocytes in a scattered manner. The scale bars for **(A-B)** are 100 μm, for **(C-D)** are 50 μm. ★ indicates tumor necrosis.

All the biopsy samples of recurrent tumor (n = 132) were collected. The samples were fixed by formalin and embedded into paraffin using a tissue processor. Among the 132 patients enrolled, there were 106 males and 26 females. The median time to recurrence after the initial treatment was 33.3 months (ranges from 6.6 to 190.2 months). The median age at recurrence was 46-year-old (ranges from 28 to 69 years). According to the 7^th^ edition of the UICC / AJCC system, 101 patients (76.5 %) were at III-VI stage and 32 patients (24.2 %) were lymph node positive simultaneously. Pathology proven recurrent undifferentiated non-keratinizing carcinoma (World Health Organization [WHO] III) and differentiated non-keratinizing carcinoma (WHO II) were diagnosed in 125 and 7 patients, respectively. The median HB level before salvage therapy was 139.0 g/l (ranges from 101.0 to 171.0 g/l) for all patients, among which 32 patients were anemic (HB < 130 g/l in men; < 120 g/l in women). Tumor necrosis before retreatment presented in 49 out of 132 (37.1 %) patients by pathologic diagnosis. Representative tumor necrosis with PD-L1 staining in recurrent NPC were shown in Figure 1. The average of follow-up time was 38.6 months (ranges from 3.5 to 182.1 months).

A total of 87 out of 132 (65.9 %) patients died during follow-ups. Of these, 35 out of 87 (40.2 %) died due to severe adverse effects, including 16 out of 35 (45.7 %) from mucosa necrosis or massive hemorrhage, 19 out of 35 (54.3 %) from other radiation-related injuries. In addition, 29 out of 87 (33.3 %) patients died due to local-regional failures and 16 out of 87 (18.4 %) due to distant failures. Other causes responsible for 2 out of 87 (2.3 %) deaths included 1 cases of cardiac disease, 1 case of digestive diseases. 5 out of 87 (5.7 %) patients died due to unknown reasons. The 3-year OS rates were 56.1 % in the 132 recurrent patients. Characteristics of the enrolled patients were summarized in Table 1.

**Table 1:** Clinic-pathologic variables and immune-activity status of PD-1 and PD-L1 in 132 patients with recurrent NPC

Variables		n	PD-1positive	P value^1^	PD-L1High	P value^1^
**Gender**	Male	106	41 (38.7%)	0.702	68 (64.2%)	0.216
	Female	26	9 (34.6%)		20 (76.9%)	
**Age at recurrence (years)**	≤ 50	87	32 (36.8%)	0.718	58 (66.7 %)	1.000
	> 50	45	18 (40.0%)		30 (66.7%)	
**Time to recurrence (months)**	≤ 24	44	13 (29.5%)	0.163	26 (59.1%)	0.192
	> 24	88	37 (42.0%)		62 (70.5%)	
**Smoking status**	Smoker or ex-smoker	52	22 (42.3%)	0.398	34 (65.4%)	0.801
	Non-smoker	80	28 (35.0%)		54 (67.5%)	
**Family history**	Yes	28	13 (46.4%)	0.293	17 (60.7%)	0.452
	No	104	37 (35.6%)		71 (68.3%)	
**Histotype classification (WHO)**	II	7	1 (14.3%)	0.186	5 (71.4%)	0.784
	III	125	49 (39.2%)		83 (66.4%)	
**r-T classification**	rT1-2	31	14 (45.2%)	0.339	13 (41.9%)	0.001**
	rT3-4	101	36 (35.6%)		75 (74.3%)	
**r-N category**	N0	100	37 (37.0%)	0.713	66 (66.0%)	0.774
	N1-3	32	13 (40.6%)		22 (68.8%)	
**Clinical stage of recurrent tumour^2^**	I-II	31	14 (45.2%)	0.339	13 (41.9%)	0.001**
	III-IV	101	36 (35.6%)		75 (74.3%)	
**Tumor necrosis before retreatment**	Yes	49	18 (36.7%)	0.835	34 (69.4%)	0.610
	No	83	32 (38.6%)		54 (65.1%)	
**HB levels at local recurrence**	Anemia^3^	32	11 (34.4%)	0.639	26 (81.3%)	0.044*
	No anemia	100	39 (39.0%)		62 (62.0%)	
**Salvage treatments**	RT^4^ only	43	20 (46.5%)	0.651	27 (62.8%)	0.111
	RT^4^with CT^5^	80	31 (38.8%)		58 (72.5%)	
	Surgery^6^	6	2 (33.3%)		2 (33.3%)	
	Radiofrequency ablation^6^	3	2 (66.7%)		1 (33.3%)	
**Cause of deaths**	Disease progression	45	19 (42.2%)	0.794	36 (80.0%)	0.024*
	Other disease	2	1 (50.0%)		1 (50.0%)	
	Severe adverse effects	35	12 (34.3%)		26 (74.3%)	
	Unknown	5	3 (60.0%)		3 (60.0%)	

^1^According to the 7^th^ Edition of the AJCC / UICC Staging System for Nasopharyngeal Cancer. ^2^Anemia was defined according to World Health Organization criteria as hemoglobin <130 g/l in men and <120 g/l in women. ^3^Radiotherapy (RT) were proscribed to 114 patients by IMRT, 6 patients with conventional radiotherapy, 2 with stereotactic radiotherapy and 1 with brachytherapy. ^4^Combined chemotherapy (CT) were administered to 80 patients with RT. ^5^Induction chemotherapy were used in 2 out of 6 patients who received salvage surgery and 1 out of 3 patients treated with radiofrequency ablation.

### Clinic-pathologic correlations

**Figure 2:**
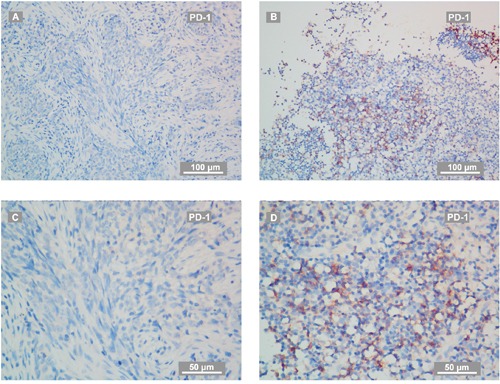
Representative IHC staining of PD-1 on recurrent NPC-biopsies **(A, C)** PD-1 staining on biopsy from a patient with recurrent NPC evaluated as PD-1 negative. The IHC photos were taken by a phase-contrast microscopy and exhibited as low **(A)** and high magnification **(C)**. **(B, D)** PD-1 staining on biopsy from a patient with recurrent NPC evaluated as PD-1 positive. The IHC photos were exhibited as low **(B)** and high magnification **(D)**. The scale bars for **(A-B)** are 100 μm, for **(C-D)** are 50 μm. PD-1+ cells were tumor-infiltration lymphocytes (TILs).

PD-1 positive immune cells presented in 50 of 132 patients (37.9%) in a scattered manner, and PD-L1 staining was detectable in 128 tumors (97.0%), which was mainly located at the membrane or in the cytoplasm region (or both) in the tumor cells. PD-L1 was also observed to be stained in tumor-infiltration lymphocytes (TILs) dispersedly. According to ROC curve analysis for OS, the optimal cut-off value of H-score was 190 (AUC: 0.702, sensitivity: 0.759, specificity: 0.511) for PD-L1. The staining of PD-L1 were considered as high level (88 cases) if H-score ≥ 190. Representative staining of PD-L1 and PD-1 in recurrent NPC were shown in Figure 1–2. Regarding the mortality of patients with high level of PD-L1 staining, there were 66 patients with high level of PD-L1 staining died during the follow-ups. Of these, 38 out of 66 patients died due to disease progression with 26 cases from local-regional failures and 12 cases from distant failures, another 28 out of 66 deaths were due to severe adverse effects, the cause of deaths were found to be significantly correlated with PD-L1 level (P = 0.024, Table 1). In this study, high level of PD-L1 staining was significantly correlated with clinical stage, rT classification of recurrent tumor and anemia at diagnose of recurrence. However, no correlation of high PD-L1 level with tumor necrosis, gender or time to recurrence was observed. No clinic-pathological parameters were found related to PD-1 positivity. Detailed data were summarized in Table 1.

### Correlation between PD-1 / PD-L1 expressions and anemia at recurrence

**Figure 3:**
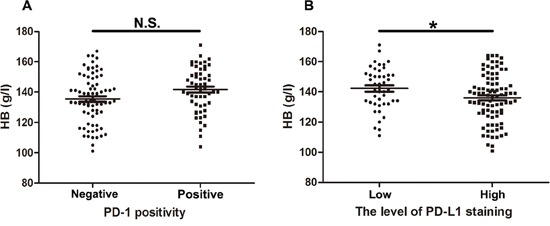
Hemoglobin in 132 NPC patients with different PD-1 / PD-L1 level **(A)** Statistical analysis suggested no significant (N. S.) difference of HB level in recurrent patients with positive or negative PD-1. **(B)** Statistical analysis revealed significant decreased HB level in recurrent patients with high level of PD-L1. T-test was used to evaluate the association of PD1/PD-L1 with HB after normality tests and homogeneity of variance test. * P < 0.05.

Pre-retreatment hematologic biomarkers such as neutrophils, lymphocytes, HB and platelets were measured to identify the potential factors related to PD-1 / PD-L1. Our result showed that no significance (N. S.) between PD-1 positivity and HB level was observed in this cohort of recurrent patients (Table 1, Figure 3A). However, anemia status at diagnoses of recurrence was negatively correlated with PD-L1 staining (P = 0.044, Table 1). Consistently, HB level was significantly reduced in patients with high level of PD-L1 staining (P = 0.029, Figure 3B).

We have also compared PD-1 / PD-L1 expression with numbers of neutrophils, lymphocytes, platelets and neutrophils / lymphocytes ratio before salvage therapy in this exploratory analysis, and no significantly relevant factors were identified (Supplementary Figure 1).

### Co-location of PD-L1 and hypoxia inducible factor-1α (HIF-1α) by double immunofluorescence analysis

**Figure 4:**
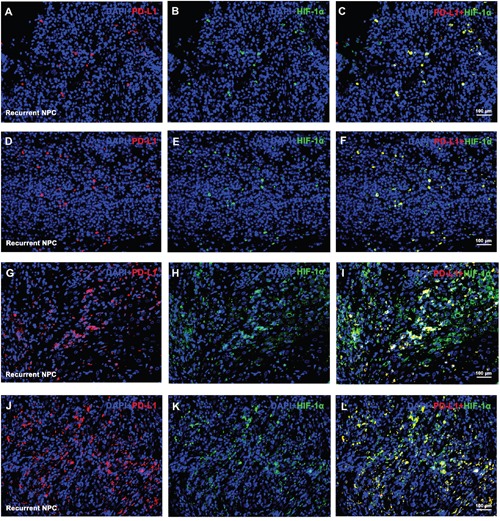
Representative double immunofluorescence staining of PD-L1 and HIF-1α on recurrent NPC-biopsies Representative immunofluorescence staining for PD-L1 (red) and HIF-1α (green) were co-located in space from 4 patients with recurrent NPC. 2 cases with relatively lower HIF-1α level **(A-C** and **D-F)** and 2 with relatively higher HIF-1α level cases **(G-I** and **J-L)** were selected as representative staining. Nuclear was stained by DAPI (blue).

Hypoxia-inducible factors (HIFs) have been demonstrated to play an important role in the regulation of erythropoiesis by coordinating a series of graded hypoxic responses [27] and stabilization of HIFs is represented as a novel therapeutic approach for the treatment of anemia [28]. Considering the closely association of HIF with anemia, immunofluorescence staining were performed to investigate the correlation of PD-L1 with anemia (represented as HIF-1α activity) using 30 recurrent tumors. PD-L1 staining was detectable in 28 cases of recurrent tumors with different extent, which was mainly distributed in the cytoplasm region of the tumor cells. HIF-1α staining was detectable in all 30 tumors, and was found to be co-located with PD-L1 staining regardless of the staining intensity or extent. Representative staining of PD-L1 and HIF-1α in recurrent NPC by immunofluorescence was shown in Figure 4A-4L. Pearson correlation coefficient of merged staining with HIF-1α and PD-L1 in Figure 4C, Figure 4F, Figure 4I, and Figure 4L were 0.94, 0.93, 0.76 and 0.93, respectively.

### Prognostic values in recurrent NPC

**Figure 5:**
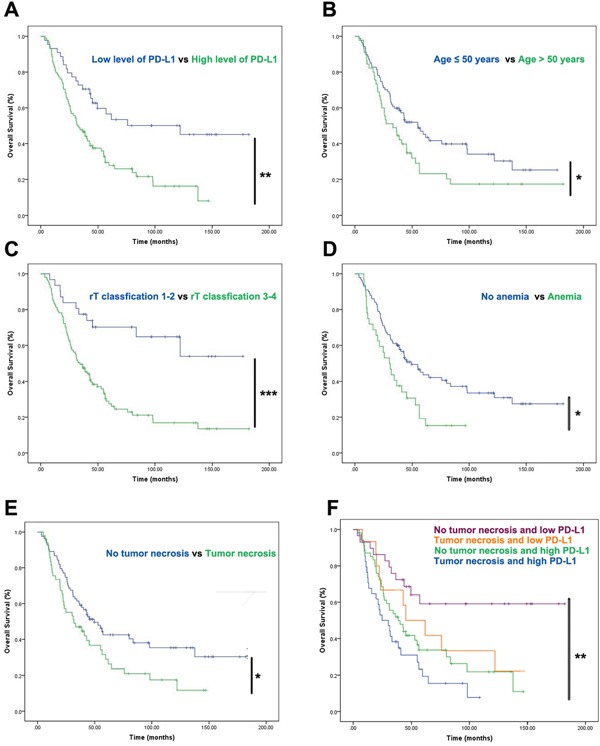
Prognostic factors of the 132 patients with recurrent NPC by Kaplan-Meier survival analysis **(A-E)** Results revealed significant links between overall survival and high level of PD-L1 **(A)**, age **(B)**, rT classification **(C)**, anemia **(D)** as well as tumor necrosis **(E)**. The cut-off points of PD-L1 were generated from ROC. **(F)** When considered as combined factors, high level of PD-L1 staining accompanied with tumor necrosis predicted the worst survival outcomes comparing with the other groups in recurrent NPC patients. Survival analysis was depicted by Kaplan-Meier method. Univariate analysis were performed with log-rank test. * P < 0.05, ** P <0.01.

To evaluate the prognostic values PD-L1 expression in recurrent NPC patients, we performed long rank tests in Kaplan-Meier survival analysis. In 132 patients with recurrent NPC, high expression of PD-L1 was correlated with significantly shorter OS (P = 0.001, Figure 5A). Factors significantly correlated with OS by univariate analyses were age at recurrence (P = 0.043, Figure 5B), the level of PD-L1 staining (P = 0.001, Figure 5A), T-stage of recurrence (P ≤ 0.001, Figure 5C), pre-retreatment anemia (P = 0.007, Figure 5D) and tumor necrosis (P = 0.026, Figure 5E). However, PD-1 positivity on TILs was not significantly correlated with OS (P = 0.950, Table 2). Further analysis by dividing patients into 4 groups regarding PD-L1 level and tumor necrosis showed that patients with both tumor necrosis and high level of PD-L1 staining in recurrent NPC had the worst survival outcomes compared with patients in other groups (P = 0.001, Figure 5F). In the multivariate analysis, significantly negative prognostic factors were identified, including age > 50 years, high level of PD-L1 staining, recurrent T3-4 stage and tumor necrosis before salvage treatment (Table 3). Moreover, patients with high level of PD-L1 might have higher risk of death (95 % CI, 0.315 - 0.888, P = 0.016, Table 3) in this cohort of 132 recurrent NPC patients.

**Table 2:** Univariate analysis of prognostic factors involved in OS with recurrent NPC

Variables		Cases(n=132)	Overall Survive rate			
			3-years	5-years	X^2^	P value^1^
**Gender**	Male	106	60 (56.6%)	27 (25.5%)	0.009	0.925
	Female	26	14 (53.8%)	7 (26.9%)		
**Age at recurrence (years)**	≤ 50	87	52 (59.8%)	26 (29.9%)	4.077	0.043*
	> 50	45	22 (48.9%)	8 (17.8%)		
**Smoking status**	Smoker or ex-smoker	52	26 (50.0%)	12 (23.1%)	1.738	0.187
	Non-smoker	80	48 (60.0%)	22 (27.5%)		
**Family history**	Yes	28	15 (53.6%)	9 (32.1%)	0.023	0.881
	N0	104	59 (56.7%)	25 (24.0%)		
**Histotype classification (WHO)**	II	7	4 (57.1%)	2 (28.6%)	0.770	0.380
	III	125	70 (56.0%)	32 (25.6%)		
**r-T classification**	rT1-2	31	24 (77.4%)	15 (48.4%)	14.698	0.000***
	rT3-4	101	50 (49.5%)	19 (18.8%)		
**r-N category**	N0	100	57 (57.0%)	28 (28.0%)	0.177	0.674
	N1-3	32	17 (53.1%)	6 (18.8%)		
**Clinical stage of recurrent tumor^2^**	I-II	31	24 (77.4%)	15 (48.4%)	14.698	0.000***
	III-IV	101	50 (49.5%)	19 (18.8%)		
**Tumor necrosis before retreatment**	Yes	49	23 (46.9%)	11 (22.4%)	4.958	0.026*
	No	83	51 (61.4%)	23 (27.7%)		
**HB levels at local recurrence**	Anemia^3^	32	13 (40.6%)	5 (15.6%)	7.200	0.007*
	No anemia	100	61 (61.0%)	29 (29.0%)		
**Time to recurrence (months)**	≤ 24	44	26 (59.1%)	8 (18.2%)	0.394	0.530
	> 24	88	48 (54.5%)	26 (29.5%)		
**Salvage treatments**	RT^4^ only	43	24 (55.8%)	13 (30.2%)	2.486	0.478
	RT^4^with CT^5^	80	43 (53.8%)	20 (25.0%)		
	Surgery^6^	6	5 (83.3%)	0 (0.0%)		
	Radiofrequency ablation^6^	3	2 (66.7%)	1 (33.3%)		
**PD-1 positivity**	Negative	82	43 (52.4%)	21 (25.6%)	0.004	0.950
	Positive	50	31 (62.0%)	13 (26.0%)		
**PD-L1 level^7^**	Low	43	32 (74.4%)	18 (41.9%)	11.701	0.001**
	High	96	42 (43.8%)	16 (16.7%)		
**PD-L1 level and Tumor necrosis**	No tumor necrosis and low PD-L1	29	22 (75.9%)	12 (41.4%)	16.606	0.001**
	Tumor necrosis and low PD-L1	15	10 (66.7%)	6 (40.0%)		
	No tumor necrosis and high PD-L1	54	29 (53.7%)	11 (20.4%)		
	Tumor necrosis and high PD-L1	34	13 (38.2%)	5 (14.7%)		

^1^Log-rank test. ^2^According to the 7^th^ Edition of the AJCC / UICC Staging System for Nasopharyngeal Cancer. ^3^Anemia was defined according to World Health Organization criteria as hemoglobin <130 g/l in men and <120 g/l in women. ^4^Radiotherapy (RT) were proscribed to 114 patients by IMRT, 6 patients with conventional radiotherapy, 2 with stereotactic radiotherapy and 1 with brachytherapy. ^5^Combined chemotherapy (CT) were administered to 80 patients with RT. ^6^Induction chemotherapy were used in 2 out of 6 patients who received salvage surgery and 1 out of 3 patients treated with radiofrequency ablation. ^7^Optimal cut-off point for PD-L1 H-score were determined by the receiver operating characteristic (ROC) curve for overall survival (OS). * P < 0.05, ** P <0.01, *** P <0.001.

**Table 3:** Multivariate analysis of prognostic factors involved in survival

Variables	Category	β	P value^1^	Hazard ratio	95% confidence interval
**Age at recurrence**	**> 50 years vs ≤ 50 years**	-0.453	0.043*	0.636	0.410-0.986
**Clinical stage of recurrent tumour^2^**	**III-IV vs I-II**	-0.988	0.003**	0.372	0.193-0.717
**Tumor necrosis before retreatment**	**Yes vs No**	-0.437	0.045*	0.646	0.422-0.989
**HB levels at local recurrence**	**Anemia^3^ vs No anemia**	-0.329	0.182	0.720	0.444-1.166
**PD-L1 level^4^**	**High vs Low**	-0.637	0.016*	0.529	0.315-0.888

^1^Cox regression analysis. ^2^According to the 7^th^ Edition of the AJCC / UICC Staging System for Nasopharyngeal Cancer. ^3^Anemia was defined according to World Health Organization criteria as hemoglobin <130 g/l in men and <120 g/l in women. ^4^Optimal cut-off point for PD-L1 H-score were determined by the receiver operating characteristic (ROC) curve for overall survival (OS). * P < 0.05, ** P <0.01.

## DISCUSSION

The immunosuppression effect of PD-1 / PD-L1 and its prognostic value in various cancers are currently a research hotspot [25]. PD-L1 inhibits T cell-mediated antitumor immunity after engagement by PD-1 on TILs [29]. Recent studies revealed that PD-L1 expression on tumor cells might predict poor prognosis in most epithelial-originated cancers [30], suggesting an effect of PD-L1 on inducing tumor progression through regulation of antitumor immunity [31]. Recurrent NPC has unique biological characteristics and poor prognosis after salvage treatment. However, the prognostic significance of PD-L1 as well as PD-1 in the recurrent NPC has not been clarified yet.

We collected biopsies of recurrent tumor and clinical data from 132 recurrent NPC patients, to analyze the expression profiles of PD-L1 / PD-1 and related factors. Our results revealed rT classification was positively correlated with the level of PD-L1 staining, indicating that the aggressive behavior of recurrent NPC with advanced stage could be partially related to immune escape induced by PD-L1. Correspondingly, patients with high level of PD-L1 had a significantly reduced overall survival (Figure 5A), which was in consist with our previous study [16] and study conducted by Zhang *et al* [17] regarding primary NPC, indicating that PD-L1 might be a potential prognostic biomarker for both primary and recurrent NPC patients. Furthermore, we analyzed the correlation between positivity of PD-1 and survival outcomes of recurrent NPC patients and found no significance available (Table 1). In primary NPC, a previous study suggested a poor prognosis of OS in primary NPC patients with PD-1-positive CD8 T cells (total n = 46) [19]. However, a larger cohort (total n = 139) demonstrated that the impact of PD-1 was not significant [17]. The prognostic value of PD-1 was largely undefined in NPC, one possible illustration might be that PD-1 expression in TILs was usually dynamically changed according to the immune status [32], and the prognostic role of PD-1 in recurrent NPC as well as in primary tumors still needs to be further clarified.

Identification of potential factors will aid in stratifying the response to anti-PD-1 blockades. Our study showed high level of PD-L1 staining was significantly correlated with anemia status at diagnose of recurrence (Table 1). Patients with high level of PD-L1 staining had significantly reduced HB levels (Figure 3B). Previous studies confirmed low baseline HB level is an adverse prognostic factor in patients with locally advanced head and neck squamous cell carcinoma [33] and NPC [34], by inducing treatment resistance via enhancing tumor hypoxia [35]. Additionally, this exploratory study revealed the co-location of PD-L1 with HIF-1α, the latter of which was known as a central element in the response to hypoxia [36]. Recent studies confirmed that hypoxia via HIF-1α might selectively up-regulate PD-L1 on immunosuppressive cells [26] and solid tumors such as pulmonary pleomorphic carcinoma [37], and oral squamous cell carcinoma [38], which contributed to the evidence for hypoxia induced immune deficiency by PD-1 / PD-L1 axis [39]. Following studies confirmed that upregulation of PD-L1 expression induced by hypoxia was mediated by STAT3 signaling pathway in anaplastic lymphoma kinase (ALK) positive pulmonary adenocarcinomas [40]. While the potential molecular mechanism behind PD-L1 and hypoxia in recurrent NPC remains unclear and requires further investigation.

Our study revealed the prognostic role of age at recurrence, PD-L1 level, anemia, tumor necrosis and rT classification in 132 recurrent NPC patients by log-rank test. Consistently, age at recurrence, rT classification, volume of recurrent tumor, response of re-radiotherapy, as well as significant complications were reported to have prognostic role in recurrent NPC patients as demonstrated by previous studies [6, 41]. In this study, we showed that tumor necrosis was correlated with worse outcomes in recurrent NPC, which was consistent with previous works [42, 43]. Notably, further analysis showed the patients with both tumor necrosis and high level of PD-L1 staining in recurrent NPC had worst survival outcomes, whereas, those patients with no tumor necrosis and low level of PD-L1 staining had better prognosis. In consist with our findings, a former study found that PD-L1 accompanied by HIF-1α surrounding necrosis in oral squamous cell carcinoma predicted a worse outcome [38]. The exact mechanism underlying the formation of tumor necrosis has yet to be fully elucidated. Pathologic changes might occur in the early stage of necrosis [43]. It has been reported that tumor necrosis often occurred in recurrent tumors due to hypoxia, hypovascularity and hypocellularity which were induced by the first course of radiotherapy [44]. In our study, anemia was not significantly correlated with tumor necrosis (P=0.186, Chi square test), indicating the complex process besides anemia in forming a tumor necrosis. These findings from our study may have implications for the future design of randomized clinical trials regarding to the application of PD-1 / PD-L1 pathway blockades in the treatment of the recurrent NPC.

In conclusion, PD-L1 and PD-1 are widely expressed in recurrent NPC tissue. PD-L1 level, age at recurrence, re-stage and tumor necrosis were prognostic factors for recurrent NPC patients. In addition, Advanced re-stage and anemia might represent as candidate biomarkers for evaluating patients’ response to anti-PD-1 / PD-L1-treatment. Further studies with a larger size of samples involved are needed to confirm these observations. Moreover, more researches are warranted to identify the molecular mechanism of the interaction between hypoxia and PD-L1 in recurrent NPC.

## MATERIALS AND METHODS

### Ethics statement

All data collection and statistical analysis of this study were conducted in accordance with the Institutional Review Board of Sun Yat-Sen University Cancer Center. Informed consent was obtained from all subjects before their salvage treatment. Individual informed consent was waived based on the Ethical Board requirement for retrospective clinical studies. Patient records / information was anonymized and de-identified prior to analysis.

### Patients and samples

A total of 179 patients with locally recurrent NPC from Sun Yat-Sen University Cancer Centre between January 2001 and March 2013 were enrolled. The cases were selected based on the following criteria: (1) local recurrence was histologically confirmed with available biopsy specimens; (2) complete clinical data; (3) non-distant metastatic simultaneously; (4) the main treatment of the initial course was radiotherapy; (5) no other malignant diseases; (6) Karnofsky score ≥ 70; (7) received salvage treatment at Sun Yat-Sen University; (8) follow-up regularly. Patients with active hepatitis, diabetes, or who failed the follow-up requirements were excluded. Therefore, we obtained the remaining 132 cases qualified for this study. All patients with recurrent NPC were re-staged according to the 7^th^ edition of the UICC /AJCC system. Formalin-fixed and paraffin-embedded (FFPE) blocks from fiberscope biopsy of NPC were retrieved from the Department of Pathology. The related clinical data of these patients were retrospectively collected from our database or hospital records, including gender, age, smoking status, family history, clinical stage, pre-therapy laboratory counts of neutrophils, lymphocytes, HB and platelets as well as follow-up records. Tumor necrosis was defined as the necrosis of tumor tissue at diagnose of recurrence and assessed pathologically by two experienced pathologists independently. Anemia was defined according to World Health Organization criteria as hemoglobin < 130 g/l in men and < 120 g/l in women. All patients were followed-up for at least 3 years.

### Salvage treatment

According to the discretion of attending oncologists with the consideration of disease condition and the preference of patients, most of these recurrent patients (114/132, 86.4 %) were given salvage IMRT, 6 patients were treated with conventional radiotherapy, 2 with stereotactic radiotherapy, 1 with brachytherapy and 3 radiofrequency ablation, salvage endoscopic nasopharyngectomy were performed for 6 patients according to the procedure previously reported by Chen MY *et al* [45]. The prescribed doses of IMRT to the recurrent tumors were 60-70 Gy in 27-35 fractions according to the institutional protocol delineated by Tian YM *et al* [41]. Cisplatin-based induction, concurrent or adjuvant chemotherapy with radiotherapy was administered to 78 patients with rT3-4 and / or bulky gross tumors. The groups included 29 patients with concurrent chemo-radiotherapy, 32 patients with induction chemotherapy followed by radiotherapy, 13 patients with induction and concurrent chemotherapy, 4 patients with adjuvant chemotherapy. Besides, 2 out of 6 patients who received salvage surgery and 1 out of 3 patients treated with radiofrequency ablation were administered with induction chemotherapy. 13 patients (9.8 %) received targeted agents (cetuximab, nimotuzumab or bevacizumab) combined with their other treatment schedules.

### Follow-up

All patients were required to be followed-up every 3-4 months in the first 3 years, every 6 months for 2 additional years, and annually after their salvage treatment. Each follow-up visit included physical examination, indirect nasopharyngoscopy, MRI of the head-and-neck regions, CT of thoracic region and ultrasound or CT of the abdomen.

### FFPE

The samples were fixed by 4 % paraformaldehyde containing 2 % sucrose in PBS at 4 °C for overnight and embedded into paraffin using a tissue processor (EG1150, Leica, Germany). FFPE sections (3 μm thick) were cut with a rotation microtome (RM2255, Leica, Germany).

### Immunohistochemistry staining

Paraffin sections were dewaxed and rehydrated by alcohol series, and then were treated with 3% H_2_O_2_ for 10 min at room temperature. The sections were steam heated for 2.5 min in ethylene diamine tetra acetic acid buffer (PH = 8.0) to retrieve antigen. Subsequent staining was performed with a 50 min incubation period at 37°C with the monoclonal antibodies PD-1 and PD-L1 (Supplementary Table 1). The immunostaining of PD-1 and PD-L1 proteins was performed on two different slides. Tonsils tissue was used as a positive control. Immunoreaction was visualized by a Peroxidase / DAB kit (Cat. K5007, Dako, Denmark). Images were taken with a phase contrast microscope (Eclipse 80i, Nikon, Japan).

### Evaluation standard of IHC

Percentages of PD-L1 positive tumor cells and PD-1 positive TILs and staining intensity were evaluated by two experienced pathologists independently who remained blind to clinical information by counting 20 sequential high-power fields (0.54 mm field diameter). Staining intensity was scored by the criteria: 0 as negative or trace, 1 as weak, 2 as moderate and 3 as high. The percentage of positive cells (0 - 100 %) was multiplied by the dominant intensity score of staining ranging from 0 to 3. Therefore, the overall semi-quantitative score, that is H-score, was ranged from 0 to 300 (maximum value of 300 corresponding to 100 % of tumor cells for PD-L1 or TILs for PD-1 staining with an overall staining intensity score of 3).

### Immunofluorescence staining

Paraffin sections were rehydrated and steam heated to retrieve antigen under the same conditions as in IHC procedure, and blocked in normal goat serum for 30 min at room temperature. The sections were incubated with primary antibodies against PD-L1 and HIF-1α (Supplementary Table 1), followed by addition of Alexa-Fluor -555 or Alexa-Fluor-488 labeled secondary antibodies (Supplementary Table 2) respectively in humidified atmosphere. Nuclei were labeled with DAPI in a concentration of 2 μg / ml (Supplementary Table 2). Sections were analyzed by a fluorescence microscope (Olympus, Cat. #BX53). Immunofluorescence analysis was performed by two experienced pathologists independently. Pearson's correlation coefficient was performed by Image-Pro plus 6.0 to evaluate the co-location of PD-L1 with HIF-1α. 0 indicates no significant correlation, -1.0 indicates complete negative correlation, and 1.0 indicates complete positive correlation.

### Statistical analyses

Optimal cut-off point for PD-L1 H-score was determined by the area under the curve (AUC) of the receiver operating characteristic (ROC) curve at the highest positive likelihood ratio point for OS. PD-L1 expression was dichotomized into two groups (high and low), using a cut-off score of ≥ 190. OS was measured from the diagnosis of local recurrence until confirmed death or the date of last follow-up through April 2016.

These statistical analyses were performed with SPSS 16.0. Measurement data were presented as mean ± standard error (S.E.M) Chi square test was used to assess the level of PD-1 and PD-L1 correlated with various clinical parameters such as age, clinical stage, anemia and tumor necrosis before re-therapy. T-test was used to evaluate the association of PD1 / PD-L1 with HB, neutrophil counts and platelet after normality test (Kolmogorov-Smirnov test). Nonparametric Mann-Whitney U test was applied to evaluate the association of PD-1 / PD-L1 with lymphocyte count and neutrophil / lymphocyte ratio. Survival analysis was depicted by Kaplan-Meier method. Univariate analysis and multivariate analysis were performed with log-rank test and Cox regression analysis, respectively. P value < 0.05 was used to denote statistical significant, and all reported P values were two sided. P values were marked as * P < 0.05, ** P <0.01, *** P < 0.001, indicating different level of significance.

## SUPPLEMENTARY MATERIALS FIGURE AND TABLES



## Abbreviations

PD-1: programmed death-1
PD-L1: programmed death-ligand 1
NPC: nasopharyngeal carcinoma
HIF-1α: hypoxia inducible factor 1α
OS: overall survival
IMRT: intensity modulated radiation therapy
HB: hemoglobin
UICC: Union for International Cancer Control
AJCC: American Joint Committee on Cancer
WHO: World Health Organization
TILs: tumor-infiltration lymphocytes
ROC: receiver operating characteristic
STAT3: signal transducer and activator of transcription 3
ALK: anaplastic lymphoma kinase
FFPE: formalin-fixed and paraffin-embedded
MRI: Magnetic Resonance Imaging
CT: Computed Tomography
PET-CT: Positron Emission Tomography-Computed Tomography
IHC: Immunohistochemistry
