# Supplementary material for: PD-1 and PD-L1 expression in 132 recurrent nasopharyngeal carcinoma: the correlation with anemia and outcomes
Source: Oncotarget. 2017 Apr 19;8(31):51210–23. doi: 10.18632/oncotarget.17214 (PMC5584243; doi:10.18632/oncotarget.17214)
Supplement: Supplementary file 1 [file oncotarget-08-51210-s001.pdf]

PD-1 and PD-L1 expression in 132 recurrent nasopharyngeal carcinoma: the correlation with anemia and outcomes

SUPPLEMENTARY MATERIALS

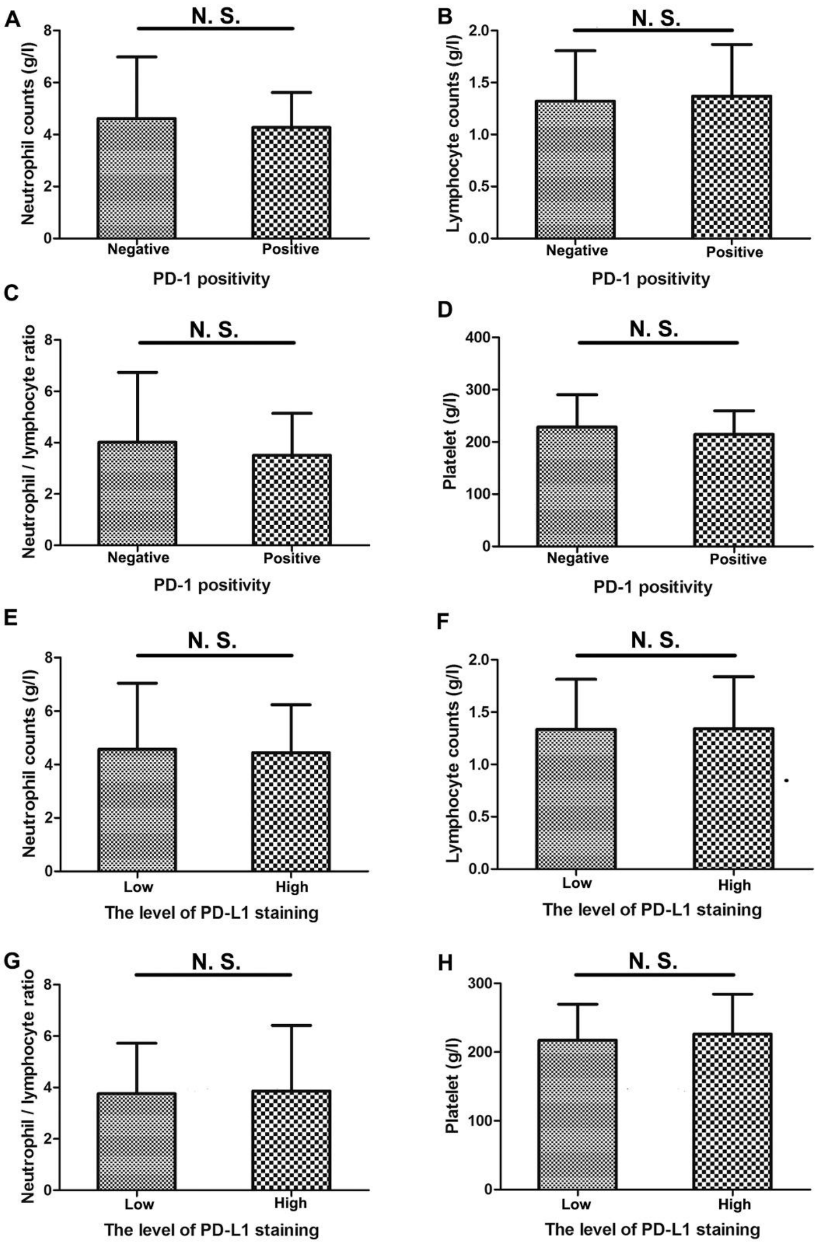

Supplementary Figure 1: Potential peripheral blood biomarkers in recurrent NPC patients with different PD-1 / PD-L1 level. **(A-D)** Statistical analysis suggested no significance (N. S.) between PD-1 positivity and pre-retreatment neutrophil counts **(A)**, lymphocyte counts **(B)**, neutrophil / lymphocyte ratio **(C)** as well as platelet **(D)** in the 132 patients with recurrent NPC. **(E-H)** Statistical analysis suggested no significance (N. S.) between PD-L1 level and neutrophil counts **(E)**, lymphocyte counts **(F)**, neutrophil / lymphocyte ratio **(G)** as well as platelet **(H)** in this cohort of recurrent patients ether. T-test was used to evaluate the association of PD-1 positivity with neutrophil counts and platelet after normality tests and homogeneity of variance test. Nonparametric Mann-Whitney U test was applied to evaluate the association of PD-1 positivity with lymphocyte count and neutrophil / lymphocyte ratio.

Supplementary Table 1: Primary and second antibodies used for immunohistochemistry (IHC) and immunofluorescence (IF) staining

Antigens	Species antibodies raised in	Methods	Dilution	Suppliers
**PD-1, human**	mouse, monoclonal	IHC	1:100	Abcam, UK, Cat. #ab52587
**PD-L1, human**	rabbit, monoclonal	IHC	1:100	Cell Signaling Technology, USA, Cat. #13684
**PD-L1, human**	rabbit, monoclonal	IF	1:250	Abcam, UK, Cat. #ab213524
**HIF-1α, human**	mouse, monoclonal	IF	1:200	Abcam, UK, Cat. #ab113642

Supplementary Table 2: Second antibodies and counterstaining of nuclei used for immunofluorescence (IF) staining

Secondary detection system used	Host	Method	Dilution	Supplier
Anti-rabbit IgG (H+L), F(ab')2 Fragment (Alexa Fluor® 555 Conjugate)	goat	IF	1:50	Cell Signaling Technology, USA, Cat. #4413
Anti-mouse IgG (H+L) F(ab')2 Fragment (Alexa Fluor® 488 Conjugate)	goat	IF	1:50	Cell Signaling Technology, USA, Cat. #4407
DAPI (nucleic acid staining)	-	IF	2µg/ml	Sigma, USA, Cat. #D9542
